# Squamous cell carcinoma malignant transformation in mature cystic teratoma of the ovary: a case report and review of the literature

**DOI:** 10.1186/s13256-024-04465-8

**Published:** 2024-03-25

**Authors:** Amir Masoud Jafari-Nozad, Najmeh Jahani, Narges Nazeri

**Affiliations:** 1 Student Research Committee, Birjand University of Medical Sciences, Birjand, Iran; 2 Department of Gynecology, School of Medicine, Valiasr Hospital, Birjand University of Medical Sciences, Birjand, Iran; 3 Department of Pathology, Faculty of Medicine, Birjand University of Medical Sciences, Birjand, Iran

**Keywords:** Mature cystic teratoma, Malignant transformation, Ovarian neoplasm, Squamous cell carcinoma

## Abstract

**Background:**

Mature cystic teratoma of the ovary is classified among the benign ovarian germ cell neoplasms, and its malignant transformation occurs very rarely (in about 2%). As a result of nonspecific signs and symptoms, preoperative diagnosis of theses malignancies is a challenge to clinicians, resulting in delayed diagnosis (in advanced stages) and poor outcomes.

**Case presentation:**

We report the case of a 43-year-old Iranian woman with progressive distension of the abdomen and hypogastric pain, who was diagnosed with squamous cell carcinoma transformation in a mature cystic teratoma of the ovary confirmed by histopathology examination. Total abdominal hysterectomy, bilateral salpingooophorectomy, and comprehensive staging surgery were performed for the patient, and she was scheduled for chemotherapy after the surgery. She responded well to the treatment and is currently continuing her chemotherapy process.

**Conclusion:**

There are a great number of reports in the literature regarding mature cystic teratoma of the ovary transformation into malignancy, so these neoplasms must be considered as a possible differential diagnosis and should be evaluated in older individuals with abdominal pain and palpable mass, or those with considerable tumor diameter and raised serum tumor markers.

## Introduction

Mature cystic teratoma of the ovary (MCTO) is a benign ovarian neoplasm consisting of well-differentiated germ cell layers. This tumor, also known as the dermoid cyst, accounts for 60% of all benign ovarian tumors [1, 2]. The etiology and pathophysiology of MCTO are not completely understood. Although some patients are asymptomatic, others usually present with abdominal pain and distention, constipation, and a noticeable abdominal or pelvic mass [3, 4]. MCTO transformation into malignant cells is rare, reported in only 1.4% of cases. Case reports indicate that those with malignant MCTO are usually between 40 and 55 years old, which is 10 years older than women with benign ovarian neoplasms [5]. Squamous cell carcinoma (SCC) is the malignant transformation most commonly reported in literature [6, 7]. SCC arising in MCTO is commonly observed in postmenopausal women [6, 8]. Tumor size, patient age, imaging findings, and elevations in serum tumor markers are some suggested risk factors associated with malignant transformation [7].

This study reports a case of MCTO in a 43-year-old women who was admitted with progressive distension of the abdomen and hypogastric pain over the previous 12 months, which was diagnosed with SCC transformation confirmed by postoperative histopathologic results. Regarding the high number of reported cases, we suggest that radiologists, pathologists, and more importantly clinicians should be aware of the possibility of MCTO malignant transformation.

## Case presentation

A 43-year-old multiparous Iranian woman, P2L2Ab1, presented to the obstetrics and gynecology department of Valiasr Hospital, Birjand, Iran in August 2023 with progressive distension of the abdomen and hypogastric pain over the previous year that had exacerbated in the past days. The pain was consistent and nonpositional. She also complained about constipation, urinary frequency, decreased appetite, and weight loss. At the initial examination, her vital signs were within normal limits. A detailed history with emphasis on gynecological condition was taken. She said that she had regular menstrual periods. The patient had no comorbidities, and there was no significant past medical history, except an ovarian dermoid cyst diagnosed 5 years previously. Since the patient was asymptotic and the cyst was smaller than 6 cm, she was not a candidate for surgery and was recommend a consistent schedule of follow-up examinations. However, she missed the appointments for an unknown reason until becoming symptomatic. The patient’s family history was insignificant in terms of any cancers or gynecological problems.

On abdominal examination, there was a palpable abdominopelvic mass in the left lower quadrant (LLQ) and hypogastric area. The mass was firm and immobile. Speculum examination revealed healthy cervix and vagina. Blood results were normal, except severe anemia (Hb: 7.5), which was treated with blood transfusion. (After transfusion of 3 units of packed cells, the patient’s Hb increased to 13.4). Serum tumor markers were assessed and reported as follows: carcinoembryonic antigen (7.1 ng/mL), alpha-fetoprotein (1 ng/mL), human epididymis protein 4 (105 pmol/L), and cancer antigen 125 (350 U/mL) (with reference ranges for CEA, AFP, HE4, and CA-125 of < 4 ng/mL, < 8 ng/mL, < 70 pmol/L, and < 35 U/mL, respectively).

Radiography was done, in which abdominal ultrasound showed large complex cystic ovarian mass, and further assessment by contrast-enhanced computed tomography (CT) revealed a large, 135 × 110 × 90 mm^3^ complex cystic lesion with fat and calcification in the pelvis with possible infiltration to lower area of the abdomen, pointing toward a possible diagnosis of ovarian mature teratoma. It should be mentioned that high-resolution CT of the lungs was normal with no signs of metastasis.

**Fig. 1:**
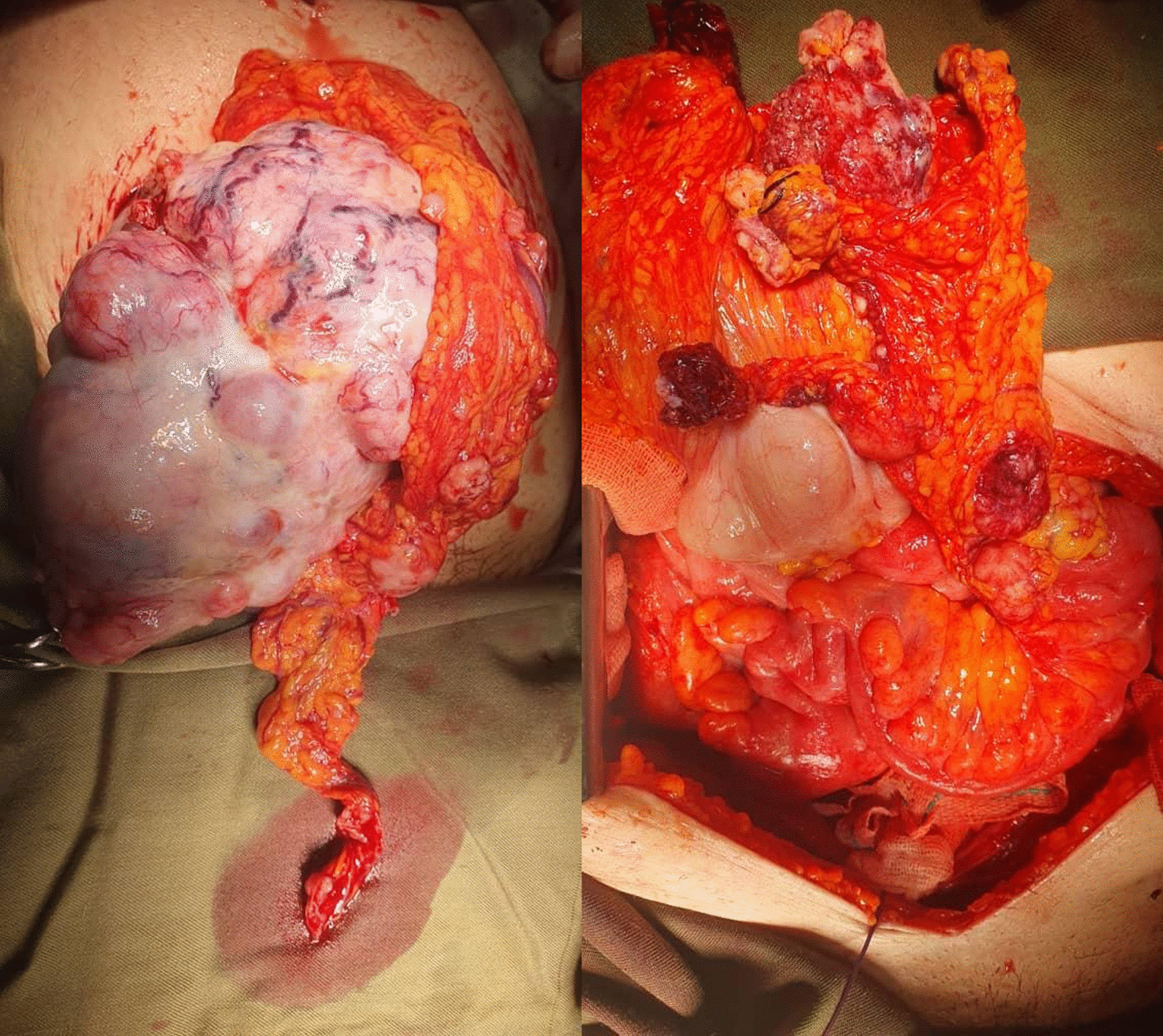
Mature cystic teratoma of the ovary with metastatic nodules (stage III)

Informed written consent was obtained, and the surgery was performed the day after admission (2 August). Total abdominal hysterectomy (TAH), bilateral salpingooophorectomy (BSO), and comprehensive staging surgery (omentectomy, appendectomy, and pelvic lymph node dissection) were performed in the standard steps [Fig. 1: mature cystic teratoma of the ovary with metastatic nodules (stage III)]. Intra- and postoperative periods were uneventful, and the patient was discharged from the hospital on 5 August.

**Fig. 2:**
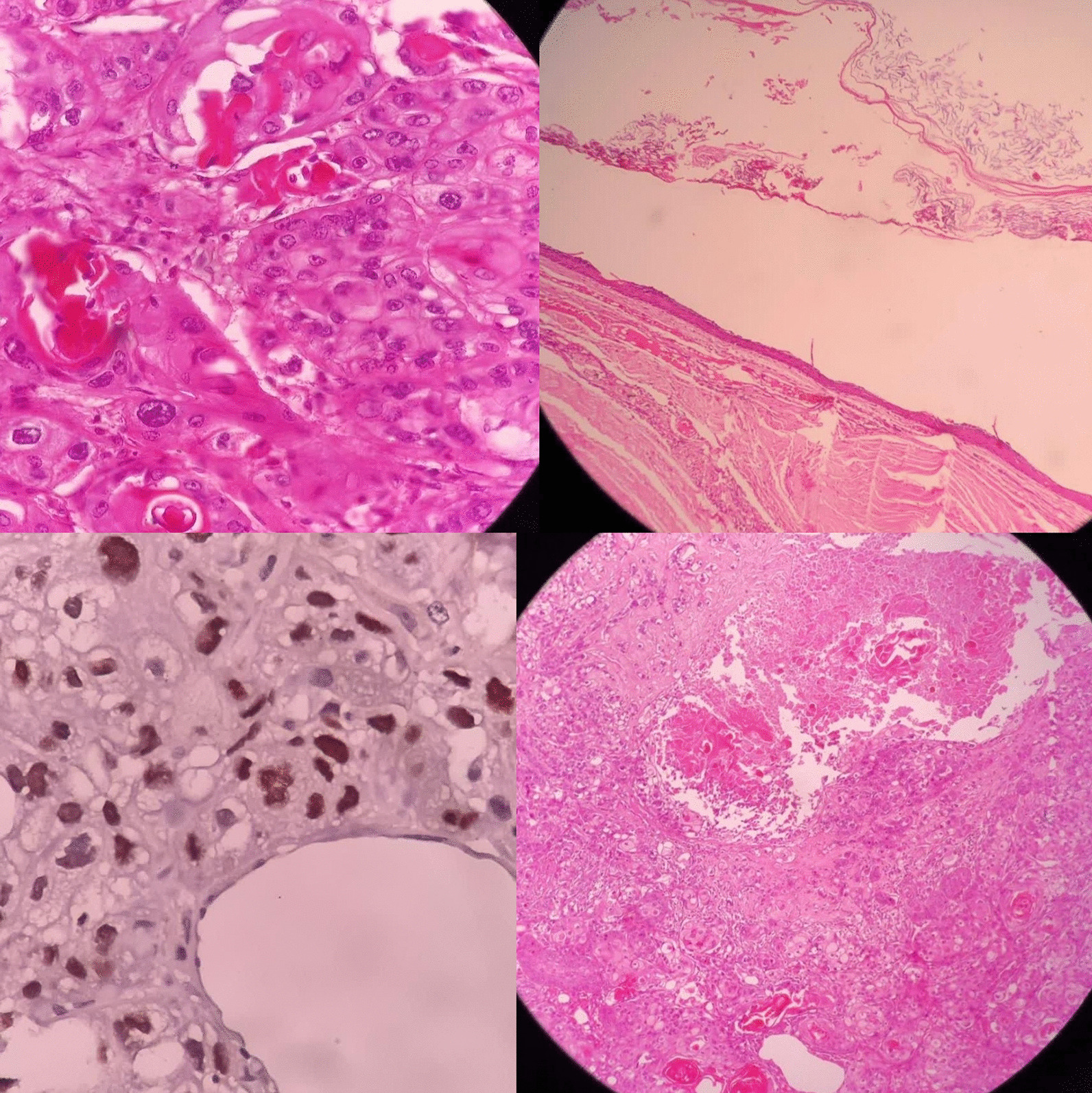
Proliferation of atypical squamous cells with keratin pearls (positive tumor protein 63 and dermoid cyst remnants with stratified squamous)

Regarding our high suspicion of malignancy before the surgery, an intraoperative frozen section was performed and the specimen was sent to the histopathology laboratory. The frozen section result showed malignancy. However, the pathological subtype could not be determined. Postoperative histopathological analysis and immunohistochemistry (IHC) findings were consistent with SCC malignant transformation of the MCTO [Fig. 2: proliferation of atypical squamous cells with keratin pearls (positive tumor protein 63 and dermoid cyst remnants with stratified squamous)]. The immunohistochemical report of epithelial markers indicated that the malignant cells were positive for P63 and CEA was positive in few tumor cells. The cells were found to be negative for CK20 and PAX8.

The patient was referred to the Iran Mehr Oncology Center for further management. Six cycles of chemotherapy with carboplatin and paclitaxel (IV) regimen every 3–4 weeks was considered for the patient. Recently, the patient has completed her chemotherapy cycles. She responded well to the treatment and experienced an uneventful clinical course with normal tumor markers during the treatment. She is currently under follow-up for any potential complications. We sculled follow-ups every 3 months for the first 2 years and every 6 months thereafter.

## Discussion

MCTO, known as a dermoid cyst, is a benign tumor and the most common germ cell neoplasm of the ovary [9]. It can occur at any age, but most cases are females of childbearing age. MCTO malignant transformation is very rare (about 2%), with most reported cases being SCC [8]. The other histological variants include thyroid carcinoma, papillary renal cell carcinoma, medulloblastoma, and intestinal-type mucinous adenocarcinoma [10]. Malignant transformation of the tissue components of MCTO occurs when the cells start proliferating at an increased rate, resulting in the development of cancerous cells [7]. The precise etiology of this malignant transformation is unknown [11, 12]. Case reports indicate that those with malignant MCTO are usually between 40 and 55 years old, which is 10 years older than women with benign MCTO [5]. The average age of malignant transformation in MCTO was reported to be 53.5 years in previous investigations [12], although limited SCC cases have been documented in women as young as 19 years old [12, 13].

Squamous transformation is reported in less than 1% of MCTO, mainly in postmenopausal women with unilateral tumors. We report the case of a 43-year-old woman with SCC transformation in an MCTO confirmed in the histopathology examination. Therefore, malignant transformation of MCTO should be suspected in women older than 40 years. It was reported that women aged more than 45 years have worse prognosis than younger patients [12]. Patients are usually asymptomatic at the beginning. Some important symptoms include abdominal pain and/or mass, altered bowel habits, urinary frequency, and weight loss [4]. Unfortunately, there are no specific clinical indications, serum markers, or imaging findings to diagnose MCTO transformation before surgery [14]. Some suggested, although nonspecific, risk factors for this condition are old age, postmenopausal state, increased tumor size and invasion, elevated levels of some tumor markers (CA125, CA19-9, and CEA), and tumor remnants [5, 6], but the only way to confirm malignant transformation is through histopathologic study.

Management of SCC malignant transformation of MCTO is challenging, but the acceptable procedures include BSO, TAH, and comprehensive staging surgery [12, 15]. Kashimura and colleagues revealed that women who received chemo- and radiotherapy instead of surgical treatment died within the first year [16]. Therefore, for those who were not optimally staged at the surgery, a second operation should be considered as full excision improves the prognosis [17]. Available evidence indicates that, in SCC malignant transformation, TAH can significantly lower the risk of mortality. Interestingly, omentectomy also increased the survival [18, 19]. Finally, it should be mentioned that early diagnosis of the disease, especially if limited to the ovaries, is accompanied by good prognosis and overall survival of 100% and 75% (at 2 and 5 years, respectively) [17, 20, 21].

There are a great number of reports in the literature regarding MCTO transformation into malignancy (Table 1), so it must be considered as a possible differential diagnosis and should be evaluated in older individuals with abdominal pain and palpable mass, or those with considerable tumor diameter and raised serum tumor markers.

**Table 1:** Review of literature regarding SCC malignant transformation in mature cystic teratoma of the ovary

Age	Presentation	Current menstrual status	Treatment/management	Follow-up	Ref.
62 years	• Progressive distension of the abdomen	Menopausal for the last 20 years	• Exploratory laparotomy • Total abdominal hysterectomy (TAH) • Bilateral salpingo-oophorectomy (BSO) • Intact capsule and infracolic omentectomy	She did not consent to receive adjuvant chemotherapy On follow-ups at 3, 6, and 12 months, she remained symptom-free with normal tumor markers and normal imaging tests	[8]
40 years	• Worsening lower abdominal pain • Palpable abdominal mass	She had not been having menstrual periods for almost 4 months	• BSO • The patient was referred to a gynecology oncology center	The patient passed away 4 months after surgery	[5]
65 years	• Severe abdominal pain • A mass detected in the LLQ of the abdomen	Menopausal for the last 15 years	• Exploratory laparotomy • Left unilateral salpingo-oophorectomy • Resection of the mass • She did not consent to radical surgery • The adjuvant chemotherapy was performed with a carboplatin regimen	At a 6-month follow-up after chemotherapy, laboratory and imaging tests were normal and the patient remained symptom free	[22]
73 years	• Acute abdominopelvic pain	Menopausal for the last 23 years	• Exploratory laparotomy • Left ovariectomy • TAH • BSO • Infracolic omentectomy • Pelvic and para-aortic node dissection • Peritoneal washing	Follow-up visits were performed every 6 months for the first 3 years and subsequently yearly. The patient died of myocardial infarction on 10 May 2021	[23]
55 years	• Lower abdominal pain • Nausea and vomiting	Not mentioned	• Exploratory laparotomy • Total abdominal uterus • Bilateral appendages • Omentectomy	She underwent four combined chemotherapy treatments of docetaxel and carboplatin, and no tumor recurrence and metastasis were seen	[24]
47 years	• Deteriorating abdominal distention • Increasing pain	Not mentioned	• Emergency laparoscopy • Left salpingo-oophorectomy	Full recovery without complications	[25]
63 years	• Lower abdominal discomfort	Not mentioned	• Laparoscopic hysterectomy • BSO • Omentectomy • Pelvic lymph node dissection • Three cycles of platinum-based chemotherapy	Patient experienced two recurrences treated with external beam radiation therapy and cycles of cisplatin plus 5-fluorouracil adjuvant chemotherapy There has been no evidence of tumor recurrence	[26]
36 years	• Short-term progressive abdominal distension • Self-touching abdominal mass	Normal menstruation	• Transabdominal excision of left ovarian cyst • TAH • Bilateral adnexectomy and pelvic lymphotomy	The combination of paclitaxel and carboplatin chemotherapy was administered every 3 weeks, and no signs of recurrence were found after follow-up	[27]
36 years	• Routine annual follow-up examinations	Normal menstruation	• Cervical cancer screening • Exploratory laparotomy • TAH • Bilateral adnexectomy • Pelvic lymph node dissection • Omentectomy • Lysis of abdominal adhesions	Not mentioned	[28]
46 years	• Abdominal distension • Menorrhagia	Not mentioned	• Exploratory laparotomy • TAH • Bilateral pelvic and paraaortic lymph node dissection • Omentectomy • Six cycles of adjuvant chemotherapy treatment (carboplatin–paclitaxel combination)	Post-treatment follow-up continues in remission	[29]
29 years	• Right lower abdominal pain • Bloating • Occasional nausea and vomiting	Normal menstruation	• Laparoscopic bilateral ovarian cystectomy • Exploratory laparotomy • Lysis of adhesions • Right salpingo-oophorectomy • Right pelvic lymphadenectomy • Omentectomy • Ileocecectomy with ileocolonic anastomosis • Radical resection of retroperitoneal tumor • Chemotherapy • Palliative external beam radiation	The patient died nine months after her initial diagnosis	[30]
73 years	• Low abdominal pain	Post menopause	• Exploratory laparotomy • TAH • BSO • Partial omentectomy • Interval debulking surgery • Chemotherapy	12 months after interval debulking surgery, no recurrence has been observed	[31]
41 years	• Lower abdominal pain • Nausea and vomiting • Fatigue	Normal Menstruation	• Exploratory laparotomy • Modified radical hysterectomy • BSO • Omentectomy • Resection of abdominal wall mass • Left pelvic lymph node dissection • Resection of left infundibulopelvic ligament • Radical tumor debulking • Six cycles of systemic chemotherapy	The patient remained on therapy, completing 25 cycles over 18 months of continuous treatment. The patient tolerated the treatment very well and resumed normal activities of daily living without assistance	[32]
38 years	• Annual medical examination	Normal menstruation	• TAH • BSO • Omentectomy • Appendectomy • Pelvic and para-aortic lymph node dissection	The patient is recovering well and is continuing chemotherapy as planned	[33]
65 years	• Intermittent lower abdominal pain for 6 months	Post menopause	• TAH • BSO	The patient did not receive any adjuvant chemotherapy and was followed up with clinical examination for 1 year, and there was no evidence of any relapse clinically	[34]

*TAH* Total abdominal hysterectomy, *BSO* Bilateral salpingo-oophorectomy, *LLQ* The left lower quadrant

## Conclusion

Malignant transformation of MCTO is a rare ovary neoplasm with challenging diagnosis and management, resulting in delayed diagnosis in advanced stages and poor outcomes. Early detection and timely management are crucial for a higher chance of successful surgical operation. We report the case of a 43-year-old woman diagnosed with SCC transformation in an MCTO confirmed in the histopathology investigation. On the basis of a review of prior reports in literature, BSO, TAH, and comprehensive staging surgery, as well as chemotherapy, is an acceptable approach to manage patients with favorable prognoses.

## Data Availability

The data that support the findings of this study are available on request from the corresponding author.
